# Health Precautions Taken by Travelers to Countries with Ebola Virus Disease

**DOI:** 10.3201/eid2205.160041

**Published:** 2016-05

**Authors:** Ifeoma Ezeoke, Alhaji Saffa, Seth Guthartz, Anna Tate, Jay K. Varma, Neil M. Vora

**Affiliations:** New York City Department of Health and Mental Hygiene, New York, New York, USA (I. Ezeoke, A. Saffa, S. Guthartz, A. Tate, J.K. Varma, N.M. Vora);; Centers for Disease Control and Prevention, Atlanta, Georgia, USA (N.M. Vora)

**Keywords:** Ebola, Ebola virus, viruses, Ebola virus disease, malaria, influenza, health precautions, travelers

**To the Editor:** To facilitate early recognition of Ebola virus disease (EVD), the New York City Department of Health and Mental Hygiene (DOHMH) actively monitored persons who had recently traveled from an EVD-affected country (*1*,*2*). Clinical manifestations of EVD are nonspecific and can resemble common travel-associated illnesses, such as malaria and influenza, both of which are potentially preventable through use of certain health precautions (*3*,*4*). Given the consequences of missing an EVD diagnosis, symptomatic persons under active monitoring who actually have non-EVD illnesses are often first isolated and tested for Ebola virus, which can delay appropriate care for the true cause of their illness and consume substantial resources. We evaluated the health precautions taken by persons traveling to EVD-affected countries.

During March 16, 2015–December 29, 2015 (the last day of EVD active monitoring by DOHMH), persons who underwent active EVD monitoring by DOHMH and who reported living in the United States for most of the previous year were asked about health precautions taken when traveling to an EVD-affected country, regardless of whether they had symptoms. Health precautions assessed were whether a healthcare provider was visited for pretravel medical advice, whether malaria prophylaxis was used during the previous 7 days (if the date of departure from the EVD-affected country was within the previous 7 days), and whether influenza vaccination was received within the past year. Health precautions were examined by country visited, sex, age, reason for travel, and citizenship. Relative risks (RRs) and 95% CIs were calculated.

During the evaluation period, DOHMH actively monitored 4,230 persons, of whom 2,032 (48.0%) reported living in the United States. Among these 2,032 persons, only 1,265 (62.3%) received pretravel medical advice and 1,198 (59.0%) received influenza vaccination. Among the 1,992 persons whose date of departure from the EVD-affected country was within the previous 7 days of the date of data collection, 822 (41.3%) used malaria prophylaxis (Table).

**Table:** Health precautions taken by 2,032 travelers to countries with Ebola virus disease who underwent active monitoring by the New York City Department of Health and Mental Hygiene after returning to the United States, March–December 29,2015*

Characteristic	Health precaution†							
	Pretravel medical advice			Malaria prophylaxis‡			Influenza vaccine in past 12 mo	
	No. (%)	RR (95% CI)		No. (%)	RR (95% CI)		No. (%)	RR (95% CI)
Country visited								
Guinea	960 (62.3)	0.91 (0.73–1.13)		**567 (37.4)**	**0.51 (0.41–0.62)**		932 (60.5)	0.89 (0.72–1.11)
Liberia	85 (57.8)	0.84 (0.65–1.09)		**71 (50.0)**	**0.67 (0.52–0.87)**		79 (53.7)	0.79 (0.61–1.02)
Sierra Leone	194 (63.4)	0.93 (0.74–1.17)		**158 (53.0)**	**0.71 (0.57–0.89)**		**161 (52.6)**	**0.77 (0.61–0.98)**
Multiple countries	26 (68.4)	Reference		26 (74.3)	Reference		26 (68.4)	Reference
Sex								
F	**574 (71.5)**	**1.27 (1.19–1.35)**		**409 (52.3)**	**1.55 (1.40–1.71)**		**561 (69.9)**	**1.35 (1.26–1.44)**
M	691 (56.2)	Reference		413 (34.1)	Reference		637 (51.8)	Reference
Age, y								
<5	**106 (82.2)**	**1.37 (1.24–1.51)**		**74 (57.4)**	**1.54 (1.30–1.83)**		**100 (77.5)**	**1.44 (1.29–1.61)**
5–14	**144 (80.5)**	**1.34 (1.22–1.47)**		**103 (57.9)**	**1.59 (1.36–1.84)**		**127 (71.0)**	**1.34 (1.20–1.50)**
15–24	82 (60.7)	1.01 (0.87–1.17)		58 (43.6)	1.16 (0.94–1.43)		75 (55.6)	1.08 (0.92–1.27)
25–44	509 (59.9)	Reference		312 (37.6)	Reference		454 (53.4)	Reference
45–64	384 (56.3)	0.94 (0.86–1.02)		254 (38.0)	1.01 (0.88–1.15)		**404 (59.2)**	**1.11 (1.02–1.21)**
≥65	40 (70.2)	1.17 (0.98–1.39)		21 (38.9)	1.05 (0.74–1.48)		**38 (66.7)**	**1.24 (1.02–1.51)**
Reason for travel								
Business	161 (61.9)	1.00 (0.91–1.11)		**140 (58.1)**	**1.54 (1.37–1.75)**		**135 (51.9)**	**0.86 (0.76–0.97)**
Education	12 (70.6)	1.13 (0.83–1.54)		7 (41.2)	1.09 (0.61–1.93)		7 (41.2)	0.68 (0.38–1.19)
Service-related§	45 (70.3)	1.13 (0.96–1.33)		**46 (78.0)**	**2.07 (1.78–2.40)**		37 (57.8)	0.96 (0.78–1.19)
Tourism	6 (33.3)	0.53 (0.28–1.03)		10 (55.6)	1.47 (0.97–2.24)		9 (50.0)	0.82 (0.52–1.30)
Visiting friends/relatives	1,030 (62.2)	Reference		613 (37.4)	Reference		1,001 (60.5)	Reference
Refused/unknown	11 (61.1)	1.10 (0.79–1.54)		6 (35.3)	0.99 (0.53–1.88)		9 (50.0)	0.92 (0.60–1.42)
Country of citizenship								
Guinea	**217 (57.6)**	**0.89 (0.82–0.98)**		**123 (33.0)**	**0.76 (0.65–0.89)**		220 (58.4)	0.96 (0.87–1.06)
Liberia	**19 (45.2)**	**0.71 (0.50–0.99)**		12 (29.3)	0.67 (0.41–1.08)		18 (42.9)	0.70 (0.50–1.00)
Sierra Leone	**59 (52.7)**	**0.82 (0.69–0.98)**		**31 (28.2)**	**0.65 (0.48–0.88)**		58 (51.8)	0.85 (0.71–1.02)
United States	865 (64.0)	Reference		574 (43.3)	Reference		813 (60.1)	Reference
Other/unknown	105 (70.5)	1.11 (0.99–1.24)		**82 (57.8)**	**1.34 (1.15–1.56)**		89 (59.8)	0.99 (0.87–1.14)
Total	1,265 (62.3)	NA		822 (41.3)	NA		1,198 (59.0)	NA

*RR, relative risk; NA, not applicable. †Persons with health precautions reported as unknown are not shown. Percentages are calculated for each row. Bold indicates statistically significant associations in which the CI does not include 1. ‡Data were included only if the date of data collection was within 7 d of the date of departure from an Ebola virus disease–affected country. §Persons who traveled for humanitarian aid, missionary, volunteer, research, or military reasons.

The most common reason for travel to an EVD-affected country was to visit friends or relatives, which was reported by 1,655 (81.4%) of 2,032 persons. Female travelers were more likely than male travelers to use each of the health precautions. Persons who traveled for business reasons (RR 1.54, 95% CI 1.37–1.75) or for service-related reasons (humanitarian aid, missionary, volunteer, research, or military reasons; RR 2.07, 95% CI 1.78–2.40) were more likely to use malaria prophylaxis than those who traveled to visit friends or relatives, although there were no differences for receiving pretravel medical advice. US citizens were more likely to receive pretravel medical advice than citizens of the 3 EVD-affected countries and more likely to use malaria prophylaxis than citizens of Guinea (RR 0.76, 95% CI 0.65–0.89) or Sierra Leone (RR 0.65, 95% CI 0.48–0.88).

In summary, persons traveling to EVD-affected countries frequently did not use major health precautions, despite federal travel warnings for EVD-affected countries and the consequences of a febrile illness developing (*5*). Our findings are notable because New York City represents >20% of all persons actively monitored for EVD in the United States (more than any other jurisdiction) (*1*). Most persons reported in this study traveled to visit friends or relatives and were less likely to use malaria prophylaxis than those who traveled for business or service-related reasons, which is consistent with previously reported data and of concern given that malaria can be a life-threatening illness (*4*). Nonetheless, a surprisingly low proportion of persons who traveled for business or service-related reasons received pretravel medical advice, used malaria prophylaxis, and received influenza vaccination. Public health agencies should work closely with organizations sending personnel abroad to improve their use of health precautions during travel. Furthermore, although most persons who traveled to visit friends or relatives received pretravel medical advice, few used malaria prophylaxis. The reason for this discrepancy deserves further evaluation.

Public health agencies should also work closely with communities whose members are likely to visit friends or relatives abroad and with medical providers caring for these communities to increase the use of travel health precautions, particularly when exceptional circumstances apply as during the EVD outbreak. Increasing the use of health precautions among persons traveling to an area for which active monitoring is recommended could directly benefit the travelers and improve the specificity of active monitoring by reducing the occurrence of malaria, influenza, and other preventable travel-associated illnesses.
